# Necrotizing* Pseudomonas aeruginosa* Community-Acquired Pneumonia: A Case Report and Review of the Literature

**DOI:** 10.1155/2017/1717492

**Published:** 2017-05-17

**Authors:** Satish Maharaj, Carmen Isache, Karan Seegobin, Simone Chang, Grant Nelson

**Affiliations:** ^1^Department of Internal Medicine, University of Florida College of Medicine, Jacksonville, FL 32209, USA; ^2^Division of Infectious Diseases, University of Florida College of Medicine, Jacksonville, FL 32209, USA; ^3^University of Miami/Jackson Memorial Hospital, Miami, FL 33136, USA

## Abstract

Lung cavities are not typically associated with community-acquired pneumonia (CAP). CAP due to* P. aeruginosa* is rare and even less commonly causes necrotizing pneumonia. We report a case of* P. aeruginosa* CAP that progressed to necrotizing pneumonia and was eventually fatal. Procalcitonin (PCT) has been well investigated in guiding antibiotic therapy (especially CAP) in adults. In this case, PCT at presentation and sequentially was negative. We discuss this caveat and present hypotheses as to the sensitivity and specificity of PCT and C-reactive protein (CRP) in these patients. To better characterize* P. aeruginosa* CAP, we undertook a review of cases indexed in PubMed from 2001 to 2016 (*n* = 9). The data reveal that risk factors for* P. aeruginosa* CAP include smoking, alcohol use, obstructive lung disease, sinusitis, and hot tub use. The route of infection for* P. aeruginosa* CAP remains unknown. One of the most interesting findings on reviewing cases was that* P. aeruginosa* CAP involves the right upper lobe in the vast majority. We suggest that when physicians in the community see patients with distinctly upper lobe necrotizing or cavitary pneumonia, they should consider* P. aeruginosa* in their differential diagnosis. Further studies are needed to clarify route of infection, role of PCT and CRP, and optimal therapy including drug and duration.

## 1. Introduction

Necrotizing pneumonia refers to consolidation with multiple small cavities and parenchymal necrosis. Lung cavities have not typically been associated with community-acquired pneumonia (CAP). They develop when bacterial lung infections progress to necrosis despite optimal medical therapy. The pathogenesis of necrotizing pneumonia is not exactly known but is believed to be tissue necrosis from an exuberant inflammatory response driven by toxins produced by an invasive pathogen and/or associated vasculitis and venous thrombosis [1].* Pseudomonas aeruginosa* is a Gram-negative bacterium that is notorious for severe hospital- acquired respiratory infections. Patients with serious pseudomonal infections often have risk factors such as being immunocompromised or on a ventilator [2]. However, CAP due to* P. aeruginosa* is rare [3] and has been infrequently known to cause necrotizing pneumonia.

## 2. Case Presentation

A 63-year-old female presented to her primary care physician with a 6-day history of fever, cough, and dyspnea. Her cough was productive of green sputum that was occasionally streaked with blood. She had mild chest discomfort but no chest pain or pleurisy at that time. Review of systems was otherwise unremarkable. Her medical history was significant for obstructive lung disease (COPD), hypothyroidism (well controlled), hypertension, and a long history of smoking (60 pack years). Two years ago, she had computed tomography (CT) scanning of the chest which showed emphysematous changes but no cavities. Despite having severe COPD, she continued to smoke daily and consume alcohol (80 units weekly). She used home nebulization for her COPD, generally utilizing sterile water, but occasionally tap water. There was no illicit drug use. She did not have any history of recent hospitalization within the preceding 90 days.

On examination, the patient was not in any distress and auscultation revealed mild wheezing. She did not want to receive any further testing at that time and was prescribed a 5-day course of levofloxacin 750 mg daily. Two weeks later, the patient presented for follow-up with worsening cough, increasing hemoptysis, and respiratory distress. On examination, she was in moderate respiratory distress with an oxygen saturation by pulse oximetry of 92% on room air, but not febrile or tachycardic, with a blood pressure of 167/97 mmHg.

**Figure 1:**
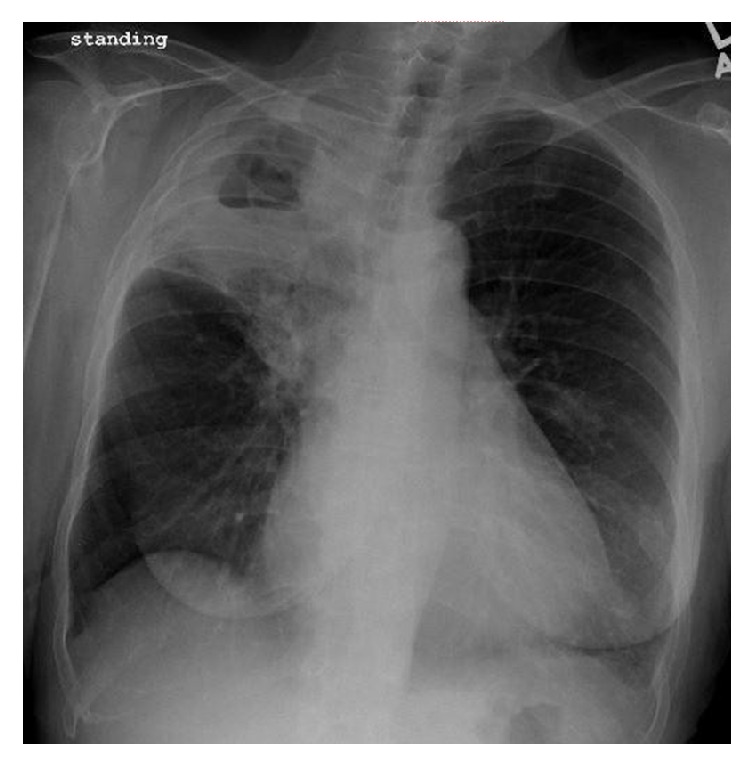
Chest X-ray with right upper lobe cavitating pneumonia.

**Figure 2:**
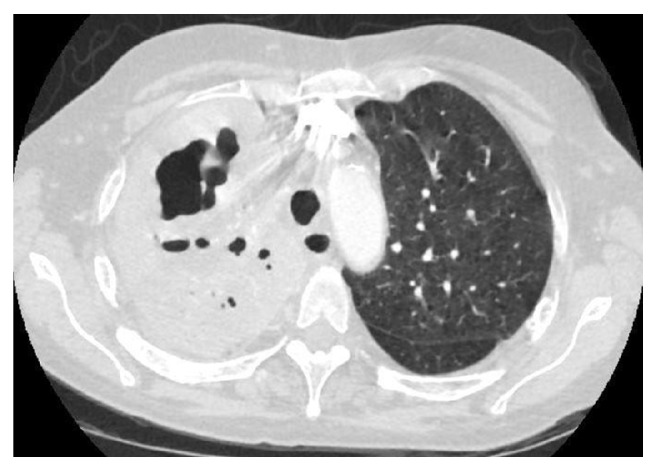
CT scan of the right upper lobe.

Chest X-ray was done revealing right upper lobe opacification with a large air-fluid level suggestive of cavitating pneumonia (Figure 1). On admission then, leukocyte count was normal at 7.8 × 10^3^/mL with no left shift; procalcitonin (PCT) was 0.12 ng/mL and C-reactive protein (CRP) was elevated at 143.8 mg/L. Testing for renal, thyroid, and hepatic function and cardiac enzymes was within normal. Autoimmune panel was unremarkable. Testing for human immunodeficiency virus (HIV), bacterial and fungal urine, and serum antigens was all negative. CT scanning confirmed extensive opacification nearly completely filling the right upper lobe (Figure 2). Also new were associated cavitary regions within the right upper lobe. The largest area of cavitation measured 5.1 × 4.2 cm.

Two sputum cultures were collected separately and Gram stain revealed no squamous epithelial cells, few neutrophils, and Gram-negative rods. Within one day, both samples grew* Pseudomonas aeruginosa*, which was resistant to levofloxacin and ciprofloxacin. The patient was started on ceftazidime, as the isolate tested susceptible to cefepime, ceftazidime, gentamicin, piperacillin/tazobactam, and tobramycin. Two days into inpatient stay, the patient developed a leukocytosis of 13.1 × 10^3^/mL with left shift. By that time, tuberculosis had been excluded with negative sputum for acid fast bacilli, along with a negative interferon-gamma release assay. Five days into admission, with no improvement, the patient underwent bronchoalveolar lavage and culture again grew* P. aeruginosa* with negative tuberculosis testing. Quantitative microbiologic analysis of the bronchoalveolar lavage was reported at 20,000 colonies/mL. The pattern of antibacterial susceptibility was exactly the same as that reported on the sputum cultures. One week into admission, the patient developed respiratory failure requiring intubation and was admitted to intensive care. Despite prolonged treatment, the patient died from progressive respiratory and multiorgan failure two weeks from admission.

## 3. Discussion and Literature Review

### 3.1. Pathophysiology

As this case demonstrates,* P. aeruginosa* CAP can progress to necrotizing pneumonia that can be fatal. Generally speaking, the progression from CAP to necrotizing pneumonia is usually rapidly progressive and these patients tend to present shortly after in acute respiratory distress. However,* P. aeruginosa* CAP appears to be an insidious disease, which can take weeks before respiratory decompensation. In the case above, we hypothesize that over the initial two weeks there would have been progressive necrosis, with coalescing of these foci into cavities which were seen on chest imaging (Figure 2). One similar case reported on pathologic findings from autopsy [4]; the affected lung showed infiltration by macrophages and neutrophils, with marked alveolar necrosis and hemorrhage. These findings can be explained as* P. aeruginosa* is invasive and can cause a thrombotic endarteritis [1].

### 3.2. Laboratory Investigations

CRP and more recently PCT have entered mainstream use in helping physicians distinguish between bacterial and nonbacterial causes of pneumonia. PCT is an acute-phase hormokine released by the parenchymal cells in patients with invasive bacterial infections [5]. PCT levels are also elevated in patients with bacteremia [6]. Research has confirmed that PCT is part of the complex innate immune response leading to an inflammatory state. Transcription and translation of PCT have been demonstrated both in organs and in macrophages as a result of bacterial infection and proinflammatory cytokine release. PCT has been well investigated in guiding antibiotic therapy by predicting the probability of a bacterial etiology of respiratory tract infection (especially CAP) in adults. A PCT cut-off of 0.50 ng/mL has been recommended to rule out bacterial infection in clinical practice [7]. Sequential PCT levels have been advocated for use in guiding antimicrobial therapy [5].

This data is interesting when applied to the case above. PCT was not elevated despite the presence of necrotizing pneumonia with clinical, radiologic, and microbiologic evidence of bacterial respiratory tract infection. Sequential PCT levels were also repeatedly negative (<0.50 ng/mL). One hypothesis is that the mechanisms of activating PCT transcription were not stimulated by localized* P. aeruginosa* necrotizing infection. The leukocyte count was surprisingly normal as well, also suggesting that the infection was relatively contained. Alternatively, the PCT cut-off may need to be lowered for detection in this population.

Studies reporting on PCT in patients with complicated parapneumonic effusions and empyema have yielded mixed results. Among 18 patients with culture-positive empyema, the sensitivity and specificity of PCT were 76% and 81%, respectively [8]. This poor discriminatory performance was even using a lower threshold of 0.19 ng/mL. In a prospective study, Doğan et al. compared 33 patients with parapneumonic effusions (including 22 empyemas) with controls (nonparapneumonic effusions). Using a cut-off level of 0.105 ng/mL, the sensitivity and specificity of PCT were 67% and 97%, respectively [9]. This case and the evidence from these studies demonstrate that PCT may remain low in localized or contained pulmonary infection, even when clinically and radiologically advanced. We suggest that despite being one of the best biomarkers for pulmonary infection, the discriminatory value of PCT in the diagnosis of localized necrotizing or cavitary pneumonia requires further investigation. We also hypothesize that the PCT cut-off of 0.50 ng/mL used to discriminate bacterial pneumonia may not be reliable in these cases to guide antibiotic therapy. Until this is clarified, a normal leukocyte count and low PCT should not falsely reassure the physician or patient as to the gravity of the illness.

These are of course issues which need to be evaluated in large scale studies. Whether these findings are dependent on bacterial etiology also remains to be investigated. Theories that may explain the low sensitivity of PCT include the absence of macrophage activation leading to TNF and the inability of the pleura to synthesize PCT. This second hypothesis deserves special mention as it has already been shown that PCT levels in pleural fluid from patients with empyema are of little discriminatory value [10].

CRP is an acute-phase protein that is synthesized by hepatocytes. It is widely available and often used to screen for infectious processes. It has been reported to be less sensitive than PCT for the detection of bacterial pneumonia and is well known to be nonspecific. However, in complicated pneumonia and localized infection, an elevated CRP level has been reported to have better discriminatory performance than PCT, in both the serum and pleural fluid [10–12], therefore, measuring CRP in cases where cavitary or necrotizing pneumonia is suspected to be of value.

### 3.3. Literature Review

Rare cases of CAP from* P. aeruginosa* have been reported since the 1960s. In what we found to be the most extensive review of* P. aeruginosa *CAP, Hatchette et al. analyzed all cases indexed in PubMed from 1966 to 2000 (*n* = 11) [13]. To better characterize* P. aeruginosa* CAP and assess for any evolution in the pattern of disease, we undertook a review of cases indexed in PubMed from 2001 to 2016 (*n* = 9). Cases were reviewed if they involved adults with no recent hospitalization, with positive identification of* P. aeruginosa* from either the sputum or blood, and clinical and radiologic confirmation of active pneumonia. These cases are summarized in Table 1.

### 3.4. Risk Factors

On review of the nine cases reported in the last 15 years, patients affected continue to be middle-aged with a mean age of 45 ± 19 years. There was no gender predisposition with five females and four males affected. Risk factors identified were smoking, alcohol use, and obstructive lung disease. Less common risk factors include sinusitis and hot tub use. The route of infection for* P. aeruginosa* CAP remains unknown. One case reported direct transmission between a mother and daughter [15], but in other cases there was no history of ill contacts. Several cases have documented exposure to contaminated aerosolized water [16, 21, 22]. It is well known that* P. aeruginosa* can contaminate even distilled water, persisting up to a year after inoculation in bottled water [23].

Our patient, along with other cases reported, had emphysematous lung changes. The pathophysiology behind* P. aeruginosa* CAP in patients with COPD is complex and remains to be elucidated. While early in emphysema there is a matched V/Q defect, over time hyperventilation develops and blood flow decreases, leading to V/Q mismatching. Combined with decreased mucociliary clearance from smoking, this can be favorable to growth of aerobic bacteria. It has been shown that a subset of patients with COPD, ranging from 4% to 15%, are colonized with a clone of* P. aeruginosa* [24, 25]. This has been likened to patterns of chronic infection resembling those observed in cystic fibrosis [25, 26]. Persistent infections can lead to symptomatic exacerbations via clonal transformation to the mucoid* P. aeruginosa* phenotype. This phenotype was grown in one case of* P. aeruginosa* CAP [20]. However, this does not fully explain the mechanism of acute* P. aeruginosa* infection in the community with resulting inflammation and necrosis.

Patients generally presented with the nonspecific symptoms of cough and dyspnea, but some reported hemoptysis and chest pain [4, 17, 19]. Similar to what Hatchette et al. reported, on presentation there was a highly variable leukocyte count with cases reporting low, normal, and high levels. Perhaps reflecting the incorporation of CRP since Hatchette et al. did their review, 4 cases reported an elevated CRP ranging from 6.6 to 38.1 mg/L [16, 17].

### 3.5. Location of Pneumonia

One of the most interesting findings reported by Hatchette et al. was that* P. aeruginosa* CAP involved the right upper lobe (RUL) in two-thirds of patients. In reviewing recent cases, this finding was reproduced with the vast majority (7/9) involving the RUL. Except one case which was not specified, all involved the upper lobes. The reason why* P. aeruginosa* CAP appears to have a predilection to involve the upper lobes has never been investigated.* P. aeruginosa* is of course an aerobic bacterium and it can be hypothesized that the upper lobes provide a more favorable environment by virtue of higher ventilation to perfusion ratios. We suggest this can be a clinical clue. When physicians in the community see patients with a distinctly upper lobe necrotizing or cavitary pneumonia they should consider* P. aeruginosa* in the differential diagnosis in the appropriate clinical scenario. A low index of suspicion can be beneficial as chest radiography may lag behind clinical status and early in the disease course necrotizing pneumonia can appear as consolidation only, leading to an underestimation of the degree of parenchymal destruction [1].

### 3.6. Prognosis and Complications

Out of the nine* P. aeruginosa* CAP cases recently reported, three were fatal highlighting the severity of this disease. Younger patients tended to do better. In fatal cases the disease course involved development of septic shock characterized by organ failure. Injury to the alveolar epithelium allows the release of proinflammatory mediators into the circulation that are primarily responsible for septic shock [27]. In cases of recovery, complications included parenchymal scarring and recrudescence requiring repeated antibiotic courses. This brings up the question of duration of treatment for* P. aeruginosa* CAP. In the cases reviewed with recovery, duration of therapy varied from 2 to 6 weeks.

## 4. Conclusion

Although CAP due to* P. aeruginosa* is rare, it can cause necrotizing pneumonia with high morbidity and mortality. Physicians must maintain a high level of suspicion as* P. aeruginosa* CAP can have an insidious course. Risk factors identified were smoking, alcohol use, and obstructive lung disease. Imaging can demonstrate coalescing of infectious foci into cavities and is recommended when there is clinical suspicion. However, chest radiography may lag behind clinical status. A review of the literature suggests that when physicians in the community see patients with a distinctly upper lobe pneumonia they should consider* P. aeruginosa* in their differential diagnosis. The biomarker PCT has been well investigated in guiding antibiotic therapy for CAP; however, we suggest that based on current evidence, PCT use in this way is not reliable in management of localized necrotizing or cavitary pneumonia. CRP appears to be more sensitive in these cases. The duration of treatment has not been well defined but a minimum of 2 weeks is reasonable. Further studies are needed to clarify the route of infection, role of PCT and CRP, and optimal therapy including drug and duration.

## Figures and Tables

**Table 1:** Recent cases of *P. aeruginosa* CAP.

Reference	Age (yrs)	Sex	Risk factors	Location of pneumonia	Treatment and outcome
(1) Crnich et al. [14]	40	M	Smoking, alcohol use, emphysema, hot tub use	Right upper and middle lobes	Amp, Cipro (6 weeks total); recovery
(2) Patel et al. [15]	83	F	Asthma	Right upper lobe	Ceft, Cipro, Tob, Pip (34 days total); recovery
(3) Huhulescu et al. [16]	49	F	Smoking, hot tub use	Left lung	Pip, Mox; multiorgan failure and death
(4) Okamoto et al. [17]	39	F	Smoking, alcohol use	Right upper lobe	Ceft, Cipro, Mero, steroids, plasmapheresis; recovery
(5) Takajo et al. [4]	50	F	—	Right upper lobe	Mero; respiratory failure and death
(6) Fujii et al. [18]	29	M	Sinusitis	Right upper lobe	Pip, Levo, Tob, Cipro; recovery
(7) Kunimasa et al. [19]	25	M	Smoking	Right upper lobe	Amp, Mero, Levo (4 weeks); recovery
(8) Gharabaghi et al. [20]	26	M	—	Left upper lobe	Oflox, Cipro (2 weeks); recovery
(9) Present case	63	F	Smoking, alcohol use, emphysema	Right upper lobe	Levo, Ceftaz; multiorgan failure and death

Amp, ampicillin; Ceft, ceftriaxone; Ceftaz, ceftazidime; Cipro, ciprofloxacin; Levo, levofloxacin; Mero, meropenem; Mox, moxifloxacin; Oflox, ofloxacin; Pip, piperacillin; Tob, tobramycin.
